# The Resistance of *Vibrio cholerae* O1 El Tor Strains to the Typing Phage 919TP, a Member of K139 Phage Family

**DOI:** 10.3389/fmicb.2016.00726

**Published:** 2016-05-18

**Authors:** Xiaona Shen, Jingyun Zhang, Jialiang Xu, Pengcheng Du, Bo Pang, Jie Li, Biao Kan

**Affiliations:** ^1^State Key Laboratory of Infectious Disease Prevention and Control, National Institute for Communicable Disease Control and Prevention, Chinese Center for Disease Control and Prevention, BeijingChina; ^2^Collaborative Innovation Center for Diagnosis and Treatment of Infectious Diseases, HangzhouChina; ^3^School of Food and Chemical Engineering, Beijing Technology and Business University, BeijingChina; ^4^Institute of Infectious Diseases, Beijing Ditan Hospital, Capital Medical University, BeijingChina

**Keywords:** *Vibrio cholerae*, bacteriophage, 919TP, K139, resistance

## Abstract

Bacteriophage 919TP is a temperate phage of *Vibrio cholerae* serogroup O1 El Tor and is used as a subtyping phage in the phage-biotyping scheme in cholera surveillance in China. In this study, sequencing of the 919TP genome showed that it belonged to the *Vibrio* phage K139 family. The mechanisms conferring resistance to 919TP infection of El Tor strains were explored to help understand the subtyping basis of phage 919TP and mutations related to 919TP resistance. Among the test strains resistant to phage 919TP, most contained the temperate 919TP phage genome, which facilitated superinfection exclusion to 919TP. Our data suggested that this immunity to *Vibrio* phage 919TP occurred after absorption of the phage onto the bacteria. Other strains contained LPS receptor synthesis gene mutations that disable adsorption of phage 919TP. Several strains resistant to 919TP infection possessed unknown resistance mechanisms, since they did not contain LPS receptor mutations or temperate K139 phage genome. Further research is required to elucidate the phage infection steps involved in the resistance of these strains to phage infection.

## Introduction

*Vibrio cholerae* is a Gram-negative bacterium and the causative agent of cholera. Seven pandemics of cholera have occurred worldwide over the last two centuries. Molecular subtyping and genome sequencing have revealed that different phylogenetic clones of *V. cholerae* O1 El Tor caused various epidemics during the seventh pandemic (Mutreja et al., 2011; Didelot et al., 2015). Subtyping, including biological subtyping and molecular subtyping methods, is necessary in microbiological studies and epidemiological investigations of pathogenic bacteria (Chattopadhyay et al., 1993; Chakrabarti et al., 2000). A Phage-Biotyping Scheme was developed for the subtyping of O1 El Tor strains and has been used in the cholera surveillance in China since the 1970s (Gao et al., 1984; Xiao et al., 2013). In the phage typing part of the scheme, five typing phages (named VP1 to VP5, respectively; Gao et al., 1984; Zhang et al., 2009; Li et al., 2013; Xu et al., 2013, 2014) are used and El Tor strains can be clustered into 32 distinct phage types (from 1 to 32) according to their lytic patterns to these five phages. In the biological typing part, El Tor strains can be grouped into 12 biotypes (from a to l) according to their biological performance in lysogenicity, susceptibility to temperate phage 919TP, sorbitol fermentation, and hemolysis (Gao, 1988; Wang et al., 2009; Xiao et al., 2013). Using the phage-biotyping scheme, El Tor strains isolated from patients in epidemics could be differentiated from those isolated from environmental samples during non-epidemic periods, and the scheme has been used in the surveillance and identification of sources of cholera outbreak.

The study of phage typing mechanisms is helpful for revealing genetic differences among the different bacterial strains with the various phage types in the environment and in epidemics, for example, resistance to phage infection may confer the strain the ability to survive in the environment, with the potential of triggering an epidemic later (Faruque et al., 2005; Jensen et al., 2006). The phenotype change from sensitivity to resistance to the phage infection always results from gene mutations. In our previous studies, we found that mutations in the receptor genes (e.g., *ompW*, core oligosaccharide genes, and *O*-antigen genes) of *V. cholerae* El Tor strains may render the strains resistant to the typing phages used in the Phage-Biotyping Scheme (Zhang et al., 2009; Xu et al., 2013, 2014). Bacteria commonly avoid phage infection using mechanisms associated with phage adsorption, injection, gene replication, assembly, and release (Hyman and Abedon, 2010; Labrie et al., 2010). Additionally, integration of temperate phage into the host bacterial genome confers superinfection exclusion by a phage, as a result of which the strains carrying the temperate phage genome in their chromosome will be resistant to subsequent infection by the same phage and phages with same immunity region.

The subtyping phage 919TP is a temperate bacteriophage used in the Phage-Biotyping Scheme in cholera surveillance in China. Some *V. cholerae* O1 El Tor strains isolated during epidemic periods are sensitive to phage 919TP and lyse, while strains isolated during other epidemic periods show resistance to this phage (Wang et al., 2007; Deng et al., 2008), suggesting that these strains may belong to different genetic clones having different genetic determinants related to 919TP infection and replication. This study was initiated to identify the genetic mutation(s) in the strains resistant to 919TP infection, to reveal the genetic variance among these epidemic El Tor strains based on their phenotype (sensitivity to 919TP), and to help understand the phage-biotyping mechanism for *V. cholerae* El Tor strains. We sequenced the genome of phage 919TP and found that it belongs to the K139 phage family (Reidl and Mekalanos, 1995; Nesper et al., 1999, 2000; Kapfhammer et al., 2002). We found that 919TP-resistant *V. cholerae* strains used different strategies to avoid infection, including superinfection exclusion, receptor mutation, and as yet unknown mechanisms.

## Materials and Methods

### Bacterial Strains, Phage, and Media

Phage 919TP, isolated from an overnight culture of *V. cholerae* O1 El Tor strain 919T, was propagated on the *V. cholerae* host strain SM6. A group of 116 O1 El Tor *V. cholerae* strains isolated in different years and regions in China (**Supplementary Table S1**), including 90 toxigenic strains (*ctxAB*^+^) and 26 non-toxigenic strains (*ctxAB*^-^), were selected for the detection of sensitivity to phage 919TP infection and identification of 919TP resistance mechanisms. El Tor strain N16961 (Heidelberg et al., 2000), whose whole genome has been sequenced and which is sensitive to phage 919TP, was also included. Unless otherwise stated, all strains were grown at 37°C in/on liquid or solid (15 g/L agar) Luria broth (LB) medium. *V. cholerae* O1 El Tor strains complemented with the phosphomannomutase (*manB*) gene were grown in media containing 100 μg/mL of ampicillin (Xu et al., 2013).

### Propagation of Phage 919TP and DNA Extraction

Five milliliters of culture of strain SM6 (3 × 10^8^ CFU/mL), mixed with phage 919TP (3 × 10^6^ PFU) were added to 500 mL of LB medium, incubated at 37°C, and shaken for 5–7 h. The mixture was centrifuged at 8470 ×*g* for 10 min to remove cell debris and then filtered through 0.22 μm pore-size filters. The filtrate was treated with DNase I (1 μg/mL) and RNase A (1 μg/mL). Solid NaCl (5.84 g/100 mL) and polyethylene glycol 8000 (10 g/100 mL) were added, and the mixture was centrifuged at 8470 ×*g*. The phage particles in the precipitate were resuspended in SM buffer (Sambrook and Russell, 2001). Phage DNA was extracted as previously described (Zhang et al., 2009). DNA was precipitated with isopropanol and dissolved in TE (10 mM Tris-HCl, 1 mM EDTA; pH 8.0) buffer solution.

### Genome Sequencing of 919TP from Phage Particles and of Its Host Strain *V. cholerae* 919T

After the DNA sample was extracted, the sample quality was analyzed by gel electrophoresis. The DNA sample was then used to construct a library following shearing of the purified DNA into smaller fragments of the desired size using a Covaris S/E210 focused-ultrasonicator. The library was constructed with average insert lengths of 500 bp according to the manufacturer’s instruction (The DNA Library Prep Reagent Set for Illumina, E6000S/L, NEB). The overhanging genomic sequences resulting from fragmentation were converted into blunt ends using T4 DNA polymerase, the Klenow fragment and T4 polynucleotide kinase. Following addition of an ‘A’ base to the 3′ end of the blunt phosphorylated DNA fragments, adapters (The NEBNext Multiplex Oligos for Illumina, E7600S, NEB) were ligated to the ends of the DNA fragments. The desired fragments were then purified following gel-electrophoresis, selectively enriched, and amplified by PCR. An index tag was introduced into the adapter at the PCR stage and a library quality test was performed. The qualified library was sequenced using the Illumina HiSeq 2000 sequencing platform. The sequence were assembled into contigs and scaffolds by using SOAPdenovo 1.04. We compared the phage 919TP genome sequence to other sequences by using Blast.

### Phage Sensitivity Detection

To determine the sensitivity of *V. cholerae* strains to phage 919TP, the strains were grown for 3–5 h in LB broth, seeded in melted 0.5% agar (50°C) and poured onto LB agar plates. A 919TP phage lysate was spotted onto the seeded soft agar and plaque development was observed after overnight incubation at 37°C.

### Detection of Spontaneous Production of Phage from *V. cholerae* Strains

In order to determine whether *V. cholerae* strains spontaneously produced 919TP (or K139 related phage), bacteria were grown overnight in LB broth at 37°C. The culture was collected by centrifugation at 12,000 rpm for 1 min and the supernatant was filtered (0.22-μm pore-size filters; Nalgene). The filtrate spotted onto soft agar plates seeded with *V. cholerae* O1 El Tor strain N16961 which is sensitive to 919TP infection. After that the plates cultured overnight to observe plaque formation.

In order to detect whether the supernatants of the strains which spontaneously produced phage contained phage 919TP (or K139 family related phages), the DNA was tested. Firstly, DNase was added into the supernatant and incubated at 37°C for 30 min to digest possible chromosomal DNA. Then the stopping solution (M6101; Promega) was added to the supernatant and incubated at 65°C for 10 min to stop the reaction. After that, the supernatant was incubated at 100°C for 10 min to release the phage genome. At last, the resulting supernatant was used as the PCR template for detection of four conserved *V. cholerae* genes encoding RecA protein (*recA*), thymidylate synthase (*thyA*), alpha subunit of DNA-directed RNA polymerase (*rpoA*), and subunit B of DNA gyrase (*gyrB*), and four phage 919TP genes encoding putative capsid portal protein (*orf15*), endonuclease subunit of putative terminase (*orf19*), putative integrase (*int*), and periplasmic protein (*glo*) using specific primers (**Table 1**).

**Table 1:** Primers used in this study.

Primer	Sequence (5′–3′)
Sequencing of K139 genome	
*int-mf*	AGTGAGTGGCAAAGGTATG
*int-mr*	GGGCGTTCTGTTTCTATT
*glo-mf*	TTTGATAAGTGGGAGAAAG
*glo-mr*	ATAATAGGCGACTGAGTGA
*cox-mf*	CACGGGTAAGTGACAAAT
*cox-mr*	GTAAATGCCAAACAACGA
*rep-mf*	GATTGCCGCTGGCCATAAAG
*rep-mr*	TAAGGAGAGGCAAACGGCAG
*orf15-mf*	GTTTTTGCAGACGGGCTCAG
*orf15-mr*	CTATGCAACCGACCCGAACT
*orf19-mf*	AAACCGCAAAGCCTCACAAG
*orf19-mr*	GCTCATCAGTTACGCTCCGA
*orf28-mf*	AAGATGATACTCGCGGCCTG
*orf28-mr*	TTCTGCATCGCGCGTTTATG
*orf35-mf*	TTAACGCAAAAGACGCAGGC
*orf35-mr*	TTGCAACGTAGTGTTGCTGC
Amplification of *V. cholerae ctxB* genes	
*ctxB-mf*	ATTTTGAGGTGTTCCATGTG
*ctxB-mr*	ATAAAGCAGTCAGGTGGTCT
Amplification of *V. cholerae* specific genes	
*thyA-mf*	ACATGGGACGCGTGTATGG
*thyA-mr*	ATATGACCACCATCAGGCTTAGC
*recA-mf*	GTGCTGTGGATGTCATCGTTGTTG
*recA-mr*	CCACCACTTCTTCGCCTTCTTTGA
*rpoA-mf*	GAACAAATCAGCACGACACA
*rpoA-mr*	CACAACCTGGCATTGAAGA
*gyrB-mf*	ATCCATTCGCAAACTTACCAT
*gyrB-mr*	TTGATCGACACGCCAGA
Primers for qRT-PCR	
*orf18-rt-mf*	ATGGGACTTGACTCCGTTCT
*orf18-rt-mr*	TGCCGTTCGTTTCCTTGT
*orf24-rt-mf*	CGAGTCGCTTACGAACATC
*orf24-rt-mr*	TCTTCCTACTTTCCAATCCC
*orf28-rt-mf*	GTGTTCCTCGGCTTACTCA
*orf28-rt-mr*	TCGTTCTTTGGCGATTTT

### Oligonucleotide Primers

To facilitate the detection of phage K139-related genome sequence in *V. cholerae* strains, primer pairs were designed to amplify eight K139 genes using PCR amplification, including *int*, *glo*, *orf15*, *orf19*, *cox* (putative regulator), *rep* (putative replication protein), *orf28* (putative endolysin), and *orf35* (putative tail fiber protein; **Table 1**). The assays were run in a 25-μL reaction mixture containing 12.5 μL of 2x *Taq* MasterMix (CW Biotech, China), 10 pmol of each forward and reverse primer, and 15–20 ng of nucleic acids under the following conditions: 94°C for 5 min; 30 cycles of 94°C for 1 min, 55–60°C for 1 min, 72°C for 1 min; and a final step of 72°C for 7 min.

### Retrieval of *wbe* Gene Cluster (*O*-Antigen Biosynthesis) Data from *V. cholerae* Sequences and *wbe*-Related Gene Complementary Plasmids

The *wbe* gene cluster sequences of 11 test El Tor strains, including two sensitive strains and nine naturally resistant strains, were retrieved from our previous sequencing analyses: genomes of strains 147, 2255, 2454, 2833, 2981, X190, and 2657 were resequenced using an Illumina HiSeq 2000 on 250-bp and 6-kb paired-end libraries in 100-fold multiplexes at BGI, China (Didelot et al., 2015). The *wbe* gene clusters were assembled, and Blast searches were carried out against the reference genome of N16961. The *wbe* gene clusters of four strains (228, 1888, 2113, and 323) were obtained by sequencing of the PCR amplification products and assembly by DNASTAR (Xu et al., 2013). The *manB* deletion mutation of *V. cholerae* strain N16961 and the complementation of mutations were obtained in our laboratory (Xu et al., 2013). The complementary plasmid pBR322-rfbT, which complemented the natural *rfbT* gene mutants (serotype Inaba strains), was also constructed in our laboratory (Liang et al., 2013).

### Transcription Analysis of the Temperate K139 Phages in the El Tor Strains

To test if the temperate K139 phages had gene transcription activity, quantitative reverse transcription PCR (qRT-PCR) was performed to detect the transcription of the K139 genes *orf18-rt, orf 24-rt*, and *orf28-rt* (**Table 1**), which were involved in phage particle morphogenesis and cell lysis. *RecA* was used as the reference. Strain 919T was used as the positive control and strain 2657, which was sensitive to 919TP and has no K139 genome, was selected as the negative control. The *V. cholerae* strains were grown in LB medium overnight at 37°C and transferred into a new LB medium (1:100). Once the cells reached an OD_600_ of 1.0, they were collected by centrifugation at 4°C and immediately processed for RNA extraction. Total RNA was extracted using the Trizol reagent (Ambion), and chromosomal DNA was removed by treatment with a TURBO DNA-free^TM^ kit (Ambion). The purity of RNA samples were verified by UV spectrophotometry and agarose gel electrophoresis, and 1 μg of total RNA was used to synthesize the cDNA with SuperScript^TM^ III reverse transcriptase (Invitrogen), random primers (TaKaRa), dNTP mixture (TaKaRa), and RNase inhibitor (TaKaRa). qRT-PCR was performed using SYBR Green (TaKaRa) on a Bio-Rad CFX96 Real-Time PCR detection system. The assays were run in a 20-μL reaction mixture containing 10 pmol of each forward and reverse primer and 2 μL of nucleic acids under the following conditions: initial denaturation at 95°C for 3 s; 40 cycles of 95°C for 5 s; and 60°C for 30 s; followed by melting curve analysis, which revealed single amplicons with an appropriate melting temperature.

### Observation of Adsorption of SYBR Gold-Stained Phage 919TP onto the Surface of Bacteria

Phage lysates with titers of at least 10^8^ PFU/mL were filtered through 0.02-μm pore-size filters (6809-5002; Waterman, Germany). The filters were washed at least five times with phosphate-buffered saline (PBS) using 1 mL of PBS to extract phage. The extracted phage samples, with titers of at least 10^9^ PFU/mL, were mixed with a SYBR gold nucleic acid gel stain stock solution (S11494; Invitrogen) in a 10,000:1 (vol/vol) mixture. This mixture was subsequently incubated for 20 min at 4°C for SYBR gold staining of the phage particles. Bacterial cultures, grown to an OD_600_ of 0.3, were mixed with the gold-stained phage (1:1, vol/vol), and incubated for 10 min at 37°C. Next, bacterial/phage mixtures were centrifuged at 6,000 ×*g* for 3 min. The precipitate was resuspended in 600 μL of PBS, recentrifuged, and resuspended in 50 μL of fresh PBS. Phage 919TP adsorbed on the surface of bacteria was examined by confocal laser scanning microscopy (CLSM; FV500; Olympus, Japan).

## Results

### Genome Sequence of 919TP and Comparison with the K139 Genome

Using the Illumina HiSeq 2000 sequencing platform, 100 Mb of genomic sequence data was generated from extracted 919TP phage genomic DNA. Linear DNA of 33,133 bp with a GC content of 48.92% was observed after assembly. The complete genomic sequence of phage 919TP was deposited in the GenBank database under the accession number KU504502. Following online sequence alignment^1^, the 919TP genomic sequence was highly homologous to *Vibrio* phages of the K139, VPUSM8, and KAPPA families. A homologous fragment was also identified in the genome of *V. cholerae* strain MJ1236 (Grim et al., 2010). The genome sequence of phage 919TP was 99% identical to that of phage K139 with 99% query coverage. Therefore, phage 919TP was considered to belong to the K139 family.

The genome of the host *V*. *cholerae* strain 919T was also sequenced. A 3,955,624 bp genome with 58 scaffolds was generated from the sequence data (402 Mbp). The GC content of the genome was 47.49%. The integrated 919TP genome sequence was extracted from the genomic sequence of *V*. *cholerae* 919T and K139 sequence was used for Blast analysis and comparison with different K139 phages. The genomic sequence was 99% identical between different phages. A single base mutation was found in the intergenic region. A small fragment deletion, and 16 single base insertions, deletions, gene internal transitions, and transversions were found in the 10 genes. Six genes contained a change in an amino acid, including changes associated with three open reading frames (Rep/Orf18/Orf30) resulting in the production of truncated proteins. Orf22 and Orf23 gene variations in three members of the K139 phage family occurred as a result of one new gene. Orf35 contains a transformation from a non-polar hydrophobic amino acid to a polar neutral amino acid (proline to serine).

**FIGURE 1:**
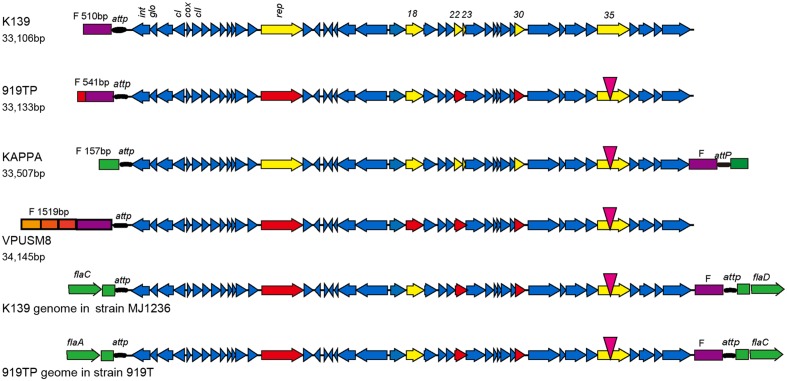
**Genome structure of phage K139 and comparison with K139-related phages.** Following sequence analysis, six genes (*rep, orf18*, *orf22*, *orf23*, *orf30*, and *orf35*) contained mutations as indicated by yellow arrows. A point mutation was observed in the *orf35* gene, indicated by an inverted red triangle. The other five genes were mutated at the head or tail of the genome as indicated by red arrows. *orf22* and *orf23* mutated together to form a new hybrid gene. Sequences of the head and end of the phages are indicated by different colors. The head sequence of the phage is indicated in violet. *V. cholerae* derived sequence is indicated in green.

Phage strain sequences (919TP, K139, VPUSM8, and KAPPA) were also compared with the phage genome sequences integrated into the lysogenic *V. cholerae* MJ1236 and *V. cholerae* 919T genomes. The comparisons showed reverse transformations between the head and tail (**Figure 1**). The F fragment in front of the attP site in the K139 and 919TP mature phage particles predominantly exists at the head of the phage genome. However, the F fragment that resulted from integration into the two *V*. *cholerae* strains shifted to generate a joint genomic tail with the attP site. Indeed, the resultant F fragment replicated in a manner similar to the whole mature phage genome by initially forming a ring, and then integrating into bacterial genomic attP locus specific sites. The genomic sequences encoding the head lengths of the analyzed phages differed in length. The K139 and 919TP phage heads were similar to each other. The head and tail sequences of the kappa phage were similar to the genome sequences of the phages integrated into the lysogenic *V. cholerae* MJ1236 and *V. cholerae* 919T genomes; the head gene fragment came from the *V*. *cholerae* sequence. The head of the VPUSM8 fragment (1,519 bp) contained three short fragments and one F fragment. The three short fragments were 100% homologous to mature phage sequences in three different positions.

The K139 phage integrated in strains MJ1236 and 919T in the same site-specific integration sequence but, interestingly, the site-specific sequence was located at different positions in the bacterial genome, between *flaC* and *flaD* in MJ1236, and between *flaA* and *flaC* in 919T.

### Strain Selection for the Analysis of Resistance Mechanisms to 919TP Infection

In this study, 116 O1 El Tor *V. cholerae* strains (**Supplementary Table S1**) were selected, including 90 toxigenic strains and 26 non-toxigenic strains. These strains were then analyzed with respect to their susceptibility to phage 919TP. Forty-three strains were found to be sensitive to 919TP, of which 36 were toxigenic strains. Subsequently, eight genes from the 919TP phage genome were selected to detect the presence of K139 phage family genes in these bacterial genomes. The eight analyzed genes were not observed in the 43 sensitive strains. Therefore, these strains were deemed not to be lysogenic, and members of the K139 phage family could infect them.

Two potential mechanisms of resistance to 919TP infection were considered in the remaining 73 resistant strains. The first mechanism was dependent on the presence of a temperate K139 genome in the bacterial host. The second mechanism involved presence of mutations in the lipopolysaccharide (LPS) biosynthesis gene cluster (Nesper et al., 2002) associated with the K139 phage receptor (Nesper et al., 2000).

### Strains Had Temperate K139 Genome Confer Resistance to 919TP Infection

The 919TP-resistant bacteria that contained the eight genes of the K139 phage family (*orf15, orf19, orf28, orf35, rep, int, cox*, and the K139 family immunity gene *glo*) were analyzed first. The eight genes could be amplified in 50 of the 73 resistant strains, suggesting that these 50 strains may harbor superinfections exclusion as they contain temperate K139 phage family DNA.

These 50 strains were tested to determine if they were capable of producing K139 phage particles. Supernatant samples of 46 of these strains generated plaques in *V. cholerae* N16961 culture, suggesting their capability for the spontaneous release of phage. All the supernatant samples from the 46 strains generating plaques on N16961 culture plates were positive for the four 919TP genes (*orf15, orf19, int*, and *glo*) but negative for the four *V. cholerae* genes (*recA, thyA, rpoA*, and *gyrB*). These results showed that the 46 strains could produce 919TP (or K139 family related) phage (**Supplementary Table S1**). The other four strains, 4070, T21, 2757, and 249, were not observed to release K139-like phage, though K139 family related genomes were present in their genomes (**Supplementary Table S1**). To test whether the genes of the temperate K139 phages in these four strains might still have the possibility to generate progeny phages, we determined the gene transcription of the temperate phages using qRT-PCR by detecting the transcription of the three phage genes *orf18* (putative major capsid protein), *orf24* (putative tail sheath protein), and *orf28*. Transcription of these genes was observed in the positive control strain 919T, and were also found in the test strains 4070, T21, 2757, and 249, suggesting that the temperate K139 phages in these strains may only generate undetectable progeny phage particles. For these strains, the possibility of defects in the progeny phage assembly at the last step of K139’s life cycle should not be excluded.

Further, the binding or adsorption of phage 919TP to temperate K139 phage family *V. cholerae* strains was analyzed by using SYBR gold-stained 919TP particles; both kinds of strains that could and could not produce detectable K139 phage were selected. Fluorescence around the bacterial cells was observed in all test strains in these experiments, indicating the binding of 919TP to these strains.

### Receptor Gene Mutations Cause Resistance to 919TP Infection in Strains that Do Not Carry the Genome of the K139 Family

**FIGURE 2:**
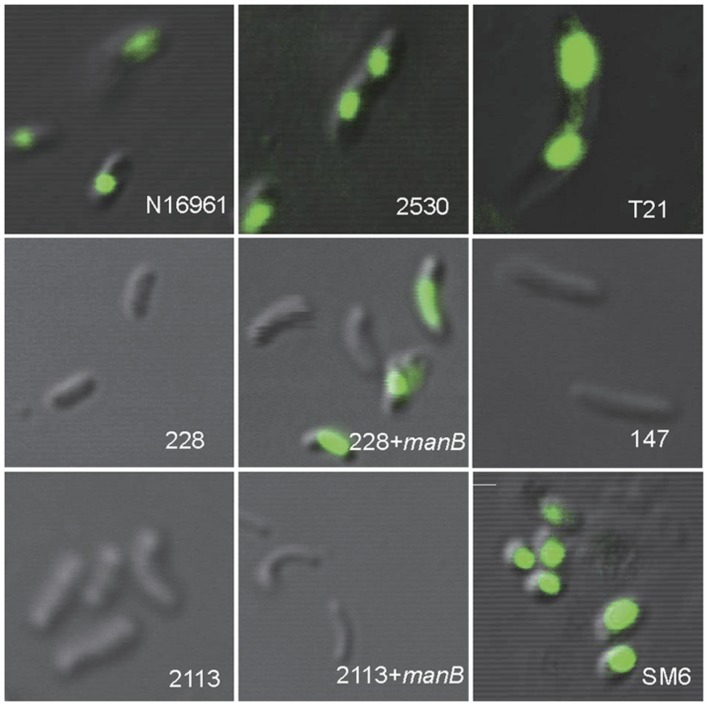
**Binding of SYBR gold-labeled phage 919TP to the surfaces of different strains of *V. cholerae* observed by confocal laser scanning microscopy.** The strains included N16961 and SM6: sensitive to 919TP; 2530: carrying temperate K139 and capable of producing detectable K139 phages; T21: carrying lysogenic K139 but incapable of producing detectable K139 phage; 228 and 2113: possessing receptor gene mutations; 228+*manB* and 2113+*manB*: complemented with the wild-type *manB*-expressing plasmid and did and did not recover sensitivity to 919TP, respectively; 147: possessing the same *wbe* gene clusters with the sensitive strain 919T but resistant to 919TP.

Twenty-three strains were 919TP-resistant but did not contain the temperate K139 phage genome (**Supplementary Table S1**). Receptor sequence mutations might have caused the failure of phage absorption to the bacterial cell, which is one resistance mechanism to phage infection. For nine of these 23 strains, whole genome sequence had been sequenced in our laboratory (Mutreja et al., 2011; Didelot et al., 2015), or the *wbe* gene cluster responsible for synthesis of the K139 phage receptor, LPS, was sequenced in our previous study (Xu et al., 2013). The *wbe* gene clusters of these nine strains, and of two other strains (323 and 2657) sensitive to 919TP, were compared with the 919TP sensitive El Tor strain N16961 (**Table 2**). Mutations in *wbe* gene clusters were found in four of the strains (**Table 2**). One (strain 323) contained only three amino acid residue substitutions in the *wbeW* gene, and this strain was sensitive to 919TP; therefore, such *wbeW* mutations should not alter 919TP phage recognition and infection and was not related to resistance to 919TP. Three of the resistant strains (228, 1888, and 2113) contained concurrent mutations in the genes *manB* and *wbeW* (**Table 2**). These strains had the same *wbeW* mutations as strain 323 that did not affect infection with 919TP. Thus, we cloned and complemented wild-type *manB* into these strains, to determine whether the *manB* mutation was involved in resistance to 919TP infection. Strain 228 became sensitive after complementation with wild-type *manB*. Moreover, CLSM showed no 919TP phage adsorption to cells of strain 228, but when complemented with wild-type *manB*, binding of 919TP to the cells was observed (**Figure 2**). Therefore, the mutations in *manB* resulted in non-binding of 919TP to strain 228, conferring resistance of the bacteria to 919TP. However, sensitivity to 919TP was not restored when strains 1888 and 2113 were complemented with wild-type *manB*. Little or no 919TP adsorption was observed to strains 1888 and 2113 in CLSM experiments (as **Figure 2**, data for strain 2113). These data suggested that in strains 1888 and 2113, there were additional resistance mechanisms to 919TP besides *manB* mutation. Here, we collected the genome data of the nine strains within these 23 919TP-resistant strains as the subgroup samples, to determine the mutations related to this phage resistance. No genome or LPS gene cluster sequence data of the other 14 resistant strains are available right now. It can be deduced that some of these strains may possess these mutations, but more receptor mutations responsible for K139 resistance or even new resistance mechanisms can be found when more resistant strains are included in the study.

**Table 2:** LPS synthesis gene cluster sequencing and associated strains.

Strain ID	Location	Source	Year isolated	*ctxB* gene	Phage production	Phage 919TP plaque formation	K139 genome sequence	LPS synthesis gene cluster mutations
228	Liaoning	Water	2004	-	-	-	-	*manB^a^, wbeW^b^*
1888	Guangdong	Water	2007	-	-	-	-	*manB^a^, wbeW^b^*
2113	Guangdong	Water	2006	-	-	-	-	*manB^a^, wbeW^b^*
147	Guangdong	Patient	2002	+	-	-	-	No mutation
2255	–	Patient	2008	+	-	-	-	No mutation
2454	Hainan	Patient	2005	+	-	-	-	No mutation
2833	Shanghai	–	1979	+	-	-	-	No mutation
2981	Shanghai	Seafood	1965	-	-	-	-	No mutation
X190	Peru	Patient	1991	+	-	-	-	No mutation
323	Liaoning	Water	2005	-	-	+	-	*wbeW^b^*
2657	Yunnan	Patient	1995	+	-	+	-	No mutation

*LPS synthesis gene cluster mutations relative to *V. cholerae* El Tor strain N16961: ^a^*manB*: Amino acid mutation in positions 72 (I to V), 77 (I to L), and 91 (Y to H). ^b^*wbeW*: Amino acid mutation in positions 13 (A to V), 118 (A to V), and 122 (S to A).*

### *V. cholerae* Strains that Were Not K139 Lysogenic Isolates and Carried the Same *wbe* Gene Clusters as that of 919TP-Sensitive Strains Were Resistant to the Phage

In the nine 919TP resistant strains for which the sequences of the *wbe* gene cluster are available, six (147, 2255, 2454, 2833, 2981, X190) had almost the same sequence as the 919TP sensitive strain 2657, except for an amino acid residue difference in *WbeT*. This gene was responsible for biotype conversion from serotype Ogawa to Inaba. However, the wild serotype Inaba strains could also be infected by 919TP, suggesting that this mutation on the *wbeT* gene, which was responsible for the serotype conversion from Ogawa to Inaba, may not generate resistance to 919TP. We introduced the plasmid expressing *rfbT* (also named *wbeT*; Liang et al., 2013) into the strains 2454 and 2833 as a representative of these six strains, and both strains recovered the agglutination ability in response to Ogawa anti-serum, indicating that the complemented *rfbT* was active, whereas neither strains showed restored sensitivity to 919TP. Therefore, this mutation in *wbeT* had no role on the resistance to 919TP infection in these strains. These six strains should possess intact LPS which could be recognized by 919TP. Furthermore, two of the strains (147 and 2255) were randomly selected to observe 919TP binding by CLSM. Unexpectedly, no fluorescence was found on the periphery of the cells, suggesting that 919TP did not bind to these strains and that there were factors other than the LPS affecting the binding of 919TP to these strains.

## Discussion

Subtyping of bacterial strains below the species level is necessary for variance and clonality studies, and to trace the waves of transmission of pandemics. Exploring the genetic determination of phenotyping can reveal the basic differences among different phenotypes. The Phage-Biotyping Scheme was used to subtype *V. cholerae* O1 El Tor strains based on their phenotype since the 1970s (Wang et al., 2007; Deng et al., 2008). Some genetic mutations of the El Tor strains that were resistant to the typing phages, mainly on the receptor genes (Zhang et al., 2009; Xu et al., 2013, 2014), had been identified. Based on phage resistance and receptor gene mutations, the appearance and spread of a distinct clone was identified in some specific geographical regions (Xu et al., 2014). *V. cholerae* strains in different epidemics or outbreaks also presented different sensitivities to phage 919TP. Therefore in this study, we explored the possible mutations of the 919TP-resistant strains and their other possible strategies by which *V. cholerae* strains resist 919TP infection, in order to reveal their genetic differences related to the 919TP infection compared to sensitive strains. This may help to understand the emergence of resistant strains and even find the genetic markers of these resistant strains.

In this study, the genome of phage 919TP was first sequenced. Unexpectedly, the phage that was in use in our Phage-Biotyping Scheme for many years was a member of the *Vibrio* phage K139 family. K139 was isolated from a *V. cholerae* serogroup O139 strain, but was frequently found in O1 El Tor strains (Reidl and Mekalanos, 1995; Nesper et al., 1999, 2000; Kapfhammer et al., 2002). Although the phage analyzed as part of this study came from *V. cholerae* strains isolated at different times and in different regions, the genome sequences of these phage strains are highly conserved.

Studying the possible strategies that *V. cholerae* strains employ to resist 919TP infection may reveal the genetic variance between the sensitive and resistant strains. As a temperate phage, lysogenicity of the phage in the host strain may confer resistance to the infection by the same phage. Phage K139 uses LPS as its receptor (Nesper et al., 2000), therefore some *wbe* gene mutations in *V. cholerae* strains may also result in resistance by hindering the binding of 919TP. Among the test 919TP resistant strains, most were K139 family lysogenic strains, which may confer superinfection exclusion by 919TP. Such superinfection did not affect the initial step of the K139 phage infection process, i.e., adsorption, but it was probably mediated at a later stage in the infection process through the inhibition of viral replication or integration of the injected genome of the newly infecting phage. Other strains were resistant because of *wbe* gene mutations that cause mutations in the K139 phage receptor thus blocking the adsorption of 919TP onto the bacteria. Therefore, in this study, the genetic characteristics (receptor mutations and temperate K139 in the 919TP-resistant strains) were directly connected to their different phenotypes (resistance to 919TP). Generally, gene mutations were easier to observe, but their biological significance was seldom identified. In the current study, mutations underlying resistance to phage infection were characterized. The biological roles of these mutations were hence identified. Such mutations may serve as markers that complement epidemiological studies implemented to distinguish and monitor the spread of *V. cholerae* strains and genetic clones.

Serotyping, biochemical tests, phage typing, molecular subtyping and whole genome sequencing are tools for the differentiation of microbiological and genetic characteristics and analysis of the evolution of microorganisms. They were also used in outbreak detection, prevalence description, and source and transmission tracing of pathogens. The Phage-Biotyping Scheme had defined the seventh cholera pandemic strains as limited phage-biotypes (Wang et al., 2007; Deng et al., 2008). The epidemic strains have undergone continuous genetic variations, for instance, mutations to obtain phage resistance conferred the strain with a survival advantage because it can avoid phage-dependent lysis. On other hand, the phage resistance phenotype and its genetic mutation can be used as the markers to distinguish the different strains, to trace the transmission of the epidemic, and to identify the source of the outbreaks, which are the purposes of the Phage-Biotyping Scheme. However, there are still some resistant strains whose resistance could not be explained by the immunity conferred by temperate phage or receptor mutations. Other possible mechanisms that were not experimentally explored in this study include the production of a structured extracellular bacterial cell matrix, degradation of phage nucleic acids through restriction-modification systems or CRISPR/Cas systems in addition to abortive infection systems (Labrie et al., 2010). Further studies are needed to examine other steps of K139 phage infection including specific adsorption, replication, and the initiation of lysis in the K139 infection cycle.

## Author Contributions

Conceived and designed the experiments: BK and XS. Performed the experiments: XS, JZ, and JX. Analyzed the data: PD and BP. Contributed reagents/materials/analysis tools: JL, JZ, and JX. Wrote the manuscript: XS. Revised the manuscript: BK.

## Conflict of Interest Statement

The authors declare that the research was conducted in the absence of any commercial or financial relationships that could be construed as a potential conflict of interest.

## Footnotes

1 http://blast.ncbi.nlm.nih.gov/Blast.cgi

## Supplementary Material

TABLE S1 **The *V. cholerae* O1 El Tor strains used in this study**.
